# Dietary Micronutrient and Mineral Intake in the Mediterranean Healthy Eating, Ageing, and Lifestyle (MEAL) Study

**DOI:** 10.3390/antiox7070079

**Published:** 2018-06-23

**Authors:** Dora Castiglione, Armando Platania, Alessandra Conti, Mariagiovanna Falla, Maurizio D’Urso, Marina Marranzano

**Affiliations:** 1 Department of Medical and Surgical Sciences and Advanced Technologies “G.F. Ingrassia”, University of Catania, 95123 Catania, Italy; doracastiglione29@gmail.com (D.C.); armplt@hotmail.it (A.P.); alessandra_conti@ymail.com (A.C.); mariagiovannafalla@yahoo.com (M.F.); 2 Provincial Health Authority of Catania, 95127 Catania, Italy; ma.durso76@gmail.com

**Keywords:** micronutrients, vitamins, minerals, Italy, population, dietary guidelines

## Abstract

Background: Dietary vitamins and minerals are essential compounds for the proper functioning of metabolic enzymes, regulation of gene transcription, and powering the body’s defense against oxidative stress. The aim of the present study was to investigate micronutrient consumption separately by age and sex, major dietary sources, and percentage of individuals meeting the recommended requirements according to Italian (Livelli di Assunzione di Riferimento di Nutrienti (LARN)) and European (European Food Safety Agency (EFSA)) agencies. Methods: Data were obtained from the Mediterranean Healthy Eating, Ageing, and Lifestyle (MEAL) study, which included a sample of 1838 individuals randomly collected in the city of Catania, southern Italy. A validated food frequency questionnaire was used to collect information on diet. Results: Intake of vitamin A, vitamin C, and vitamin B group (except vitamin B9) was in line with other reports and was adequate according to the guidelines, while the percentage of individuals meeting the guidelines for vitamin D, vitamin E, and vitamin B9 was about 3%, 10%, and 40%, respectively. Among minerals, intake of iron, magnesium, and selenium was adequate for most of the sample, while the percentage of individuals meeting the recommendations for calcium, sodium, and potassium intake was about 20%, 8%, and 35%, respectively. Conclusions: An important percentage of the population would benefit from campaigns raising awareness of micronutrient deficiency or excessive consumption potentially affecting their health.

## 1. Introduction

Adopting a healthy diet has been shown to decrease the risk of certain noncommunicable diseases, such as cardiovascular disease (CVD) and cancer [1,2]. Among the most important components of the diet, micronutrients and minerals comprise organic vitamins and inorganic trace elements necessary for homoeostasis of the body and cannot be synthetized endogenously [3]. Vitamins may serve as co-factors for many important metabolic enzymes, regulate gene transcription, and power the body’s defense against oxidative stress [4,5]. Additionally, minerals may act as co-factors for enzymatic processes and the correct functioning of body cells [6]. Altogether, vitamins and minerals are considered crucial for a healthy diet and are included in international dietary guidelines of relevant interest for patients, health-care providers, and public health policy-makers.

Among the most important vitamins, vitamin A (retinol and carotene) is important for the maintenance of epithelial cell integrity, growth, and development and participates in immune functions and normal vision [7]. Vitamin E plays a protective role against lipoproteins and polyunsaturated fatty acids (PUFA), protects cellular and intracellular membranes from damage, influences the activity of some enzymes, inhibits platelet aggregation, and is involved in erythrocyte maintenance [8]. Vitamin C is involved in the antioxidant defense system and has important implications for the immune system [9]. It is involved in the metabolism of cholesterol and in many biochemical reactions, including the synthesis of catecholamines, carnitine, and collagen [10]. Vitamin D, together with calcium and magnesium, has a relevant role in bone maintenance and development [11]. Vitamin D is a fat-soluble vitamin important in regulating serum calcium and phosphorus, iron, phosphate, magnesium, and zinc homoeostasis. Additionally, vitamin D plays an important role in cell differentiation and proliferation, and exerts beneficial effects on the immune and nervous systems. Vitamin D can be obtained either from dietary sources or by direct exposure to sunlight; under normal conditions, levels of vitamin D remain within a target range of 20–60 ng/mL. A person is in a state of vitamin D deficiency if the blood level is ≤20 ng/mL. The world is in a state of D hypovitaminosis, primarily due to less exposure to sunlight [12]. Other factors that influence vitamin D level are age, gender, ethnicity, skin color, season, clothing, and housing. Besides its effect on bone health, several observational studies suggested that vitamin D deficiency may be associated with many types of cancer, CVD, and metabolic disorders [13,14,15]. The B vitamins (thiamine (B1), riboflavin (B2), niacin (B3), pantothenic acid (B5), vitamin B6, folate (B9), and vitamin B12) are water-soluble, and they are essential for many body functions, including catabolic metabolism and anabolic metabolism. Importantly, the active forms of thiamine, riboflavin, and niacin are essential co-enzymes playing a role in mitochondrial aerobic respiration and cellular energy production, and therefore deficiency of any one B vitamin could dysregulate the aforementioned processes [16,17,18,19,20].

Among the most important minerals, dietary sodium is an essential compound responsible for maintenance of plasma volume and plasma osmolality. Nonetheless, high dietary sodium is an important risk factor for hypertension and cardiovascular and kidney disease, therefore dietary guidelines recommend that sodium intake does not exceed 2–2.4 g/day [21]. Potassium has important biological functions in neural transmission, vascular tone, and muscle contraction [22]. Zinc and selenium are essential nutrients for the antioxidant defense system. Zinc participates in many metabolic processes as a catalytic, and as a structural component and regulator of gene expression [23]. Selenium is a key component of several selenium-based proteins with essential enzymatic functions that comprise thyroid hormone metabolism, and plays a role in anti-inflammatory and anti-oxidant responses [24]. Iron is crucial for the delivery of oxygen to the cells, because it is part of hemoglobin [25]. It has important implications in antimicrobial activity exerted by phagocytes, neurotransmitter synthesis, and synthesis and function of DNA, collagen, and bile acids. Certain amounts of iron must be delivered in the diet in order to replace the iron that is lost from the body through blood loss and exfoliation of skin and gastrointestinal cells. Women, especially adolescents, who adhere to low-energy diets are at high risk for iron deficiency. Among the major food sources of iron are cereals, vegetables, nuts, eggs, fish, and meat. Iron is also added to fortified foods in many countries and is available as a supplement. Recommended iron intake for women of childbearing age is 18 mg/day, while for men and postmenopausal women it is 8 mg/day. The estimated average requirement of iron is 8.1 mg/day for fertile women, 6 mg/day for men, and 5 mg/day for postmenopausal women [26]. Magnesium is a major mineral that exists in the human body, 70% in the skeleton and the rest in the cells. Magnesium, as a constituent of chlorophyll, is contained in large quantities in green leafy vegetables. Magnesium plays an important role in muscle contraction, gland secretion, and nerve transmission, which is also required for normal cardiac electrophysiology. It also has protective effects on the cardiovascular system through enhancing endothelium-dependent vasodilation, improving lipid metabolism and profile, reducing systemic inflammation, and inhibiting platelet function [27,28]. In particular, magnesium administered with taurine lowers blood pressure, improves insulin resistance, delays atherogenesis, prevents against arrhythmias, and stabilizes platelets. In the general population, magnesium deficiency is rather common, as its intake has reduced over the years [28]. Magnesium can protect from CVD, and abnormally low circulating magnesium (<0.65 mmol/L) is a risk factor for cardiac arrest [29]. Calcium may act together with vitamin D in improving bone health, and it has been associated with a decreased risk for various types of cancers [30], despite high circulating levels possibly being a risk factor for CVD [31,32].

Better knowledge of micronutrient consumption in the Italian population is necessary to prevent and/or delay the adverse effects that result from an inadequate diet. Levels of vitamin and mineral consumption and comparisons among countries may help to characterize the level of quality of nutritional requirements of populations and identify potential gaps for healthy and proper dietary intake of such compounds. The aim of the present study was to investigate micronutrient consumption separately by age and sex, major dietary sources, and percentage of individuals meeting the recommended requirements according to Italian (Livelli di Assunzione di Riferimento di Nutrienti (LARN)) [33] and European (European Food Safety Agency (EFSA)) agencies [34].

## 2. Materials and Methods

### 2.1. Study Design and Population

The Mediterranean Healthy Eating, Ageing, and Lifestyle (MEAL) study is an observational study primarily focused on nutritional habits in a sample of individuals living in Sicily, southern Italy. The theoretical sample comprised a sample of 2044 men and women 18 years of age or older, randomly selected in the area of the city of Catania. The study enrollment was performed between 2014 and 2015 by selecting from lists of registered patients among a pool of general practitioners. Full details regarding the study protocol were published in detail previously [35]. The theoretical sample size was set at 1500 individuals to provide a specific relative precision of 5% (Type I error, 0.05; Type II error, 0.10), taking into account an anticipated 70% participation rate. Out of 2405 individuals invited to participate in the study, 2044 participants (response rate of 85%) made up the final sample. All participants were informed about the aims of the study and provided written informed consent. All the study procedures were carried out in accordance to the Declaration of Helsinki (1989) of the World Medical Association. The study protocol has been approved by the concerning ethical committee (protocol number: 802/23 December 2014).

### 2.2. Data Collection and Dietary Assessment

Electronic data collection was performed by face-to-face computer-assisted personal interviews. Demographics and health status were assessed according to standard procedures [36]. The dietary intake assessment was executed by the administration of 2 food frequency questionnaires (a long and a short version) that were previously validated for the Sicilian population [37,38]. For the purposes of this study, data retrieved from the long version was used. We used food composition tables of the Research Center for Foods and Nutrition in order to identify and calculate food intake, energy content, and micronutrient intake [39]. Intake of seasonal foods referred to consumption during the period in which the food was available and then adjusted by proportional intake during 1 year. Food frequency questionnaires (FFQs) with unreliable dietary intake (<1000 or >6000 kcal/day) were excluded (*n* = 107), leaving a total of 1838 individuals included in the final analysis.

### 2.3. Adherence to Dietary Recommendations

To investigate adherence to healthy dietary requirements for micronutrients, the European recommendations from EFSA [34] and those proposed by the Italian Society of Human Nutrition (LARN) [33] were considered for the present study.

### 2.4. Statistical Analysis

Frequencies are expressed as absolute numbers and percentages; continuous variables are expressed as means and standard errors, medians, and ranges. Differences between groups for continuous variables were calculated using Student’s *t*-test and ANOVA for continuous variables distributed normally, and Mann–Whitney U-test and Kruskall–Wallis test for variables distributed not normally. All reported P values were based on 2-sided tests and compared to a significance level of 5%. Finally, SPSS 17 (SPSS Inc., Chicago, IL, USA) software was used for all statistical analysis.

## 3. Results

Table 1 and Table 2 show the intake of vitamins and minerals in the study population, in total and separately by sex and age.

With regard to vitamins, men showed significant energy-adjusted higher intake of some vitamins from the B complex (B1, B6, B9, and B12), vitamin C, and vitamin E. When considering differences between sexes within age groups, there was significantly higher intake in men than women of vitamin B1 among younger individuals, while for vitamins B6 and B12, vitamin C, and vitamin E, there was higher intake among older individuals (Table 1). Consequently, when considering the whole sample, younger individuals (20–50 years old) had significantly higher intake of vitamin B1 and vitamin C, while among women there was significant intake of vitamin E in the same age group (Table 1). Regarding minerals, there was no significant difference in intake between sexes and age groups, with the exception of iron and potassium, which were more consumed by men than women (Table 2).

**Figure 1:**
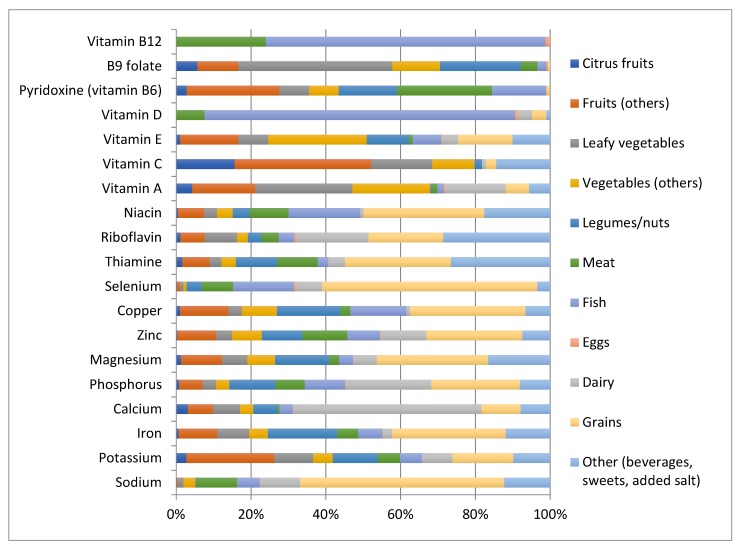
Major dietary sources of the micronutrients investigated in the MEAL study.

Figure 1 shows the major dietary sources of the micronutrients investigated in this study. Among the most interesting findings, grains were the highest contributors of selenium, but also sodium; fruits of potassium and vitamin C (especially citrus fruits); and vegetables of vitamins B9, E, and A (the latter especially from leafy vegetables). Meat also contributed sodium, while legumes and nuts contributed folate and most minerals. Fish was an important source of vitamins B12 and D, while dairy products were sources of calcium and phosphorus.

Table 3 shows the percentage of individuals meeting the EFSA’s dietary recommendations for vitamin and mineral intake. In our sample, 98% did not meet the criteria for vitamin D intake, both women and men. Among the highest adherence to the recommendations, the entire sample met the recommendations for vitamins B1 and B3, while more than half met those for iron, magnesium, selenium, zinc, and vitamins A, B2, B6, B9, B12, and C; however, more women than men met the recommendations for magnesium and zinc. In contrast, less than half of the total sample met the recommendations for calcium and potassium, while the majority did not meet the recommendations for vitamins D and E.

LARN’s dietary recommendations reflected the European ones, with more than 80% of individuals meeting dietary recommendations for sodium, magnesium, selenium, and vitamin B complex (besides vitamin B9) (Table 4). However, some differences between the sexes in iron and zinc intake were found, with recommendations for the former met by a higher percentage of men and the latter by a higher percentage of women (Table 4).

## 4. Discussion

This study provides an analysis of micronutrient intake in the MEAL cohort, a representative sample of adults from southern Italy. The main differences between sexes and age groups do not reflect any specific pattern of consumption and malnutrition among younger or older individuals. However, this report highlights alarming data concerning inadequate intake of vitamin D and vitamin E.

Women reported lower intake of certain micronutrients, which is in line with European data [40]. The same analysis showed a prevalence of inadequate micronutrient intake, especially vitamin D, vitamin C, vitamin B9, calcium, selenium, and iodine, in the adult and elderly population [40]. It has been estimated that 1 billion people worldwide suffer from vitamin D deficiency, including more than 40% of the population in the US and Europe, 82% in Italy, 70% in Korea, and about 70% in Malaysia [41]. Mean intake ranged from 1.3 mg/day in Spain to 3.5 mg/day in Poland and the Netherlands. The percentage of individuals with intake under the recommended amount ranged from 7% of men in the Netherlands to 95% of women in Spain. It is noteworthy that supplement intake or food fortification was not taken into account in this study. In other countries, especially in northern Europe, fortifying the food supply with vitamin D is mandatory [40]. Recently, it has been demonstrated by several studies that vitamin D is beneficial in the prevention of chronic diseases, and vitamin D intake is significantly associated with a reduction in the risk of osteoporosis, type 2 diabetes, cancer, multiple sclerosis, and rheumatoid arthritis [42,43]. When environmental, social, or physiological circumstances do not allow sufficient exposure to sunlight and adequate production of vitamin D, dietary compensation must occur to maintain serum 25(OH)D levels. Moreover, in those countries where the population has a diet poor in fatty fish (one of the major dietary sources of vitamin D), the only alternative way to increase exposure to natural or artificial UVB light is to fortify foods or use vitamin supplements. The major contributor of vitamin D in this sample was fish, thus the data presented in this study is further alarming, as fish consumption has been demonstrated to be high in this population [44]. Moreover, individuals with higher adherence to an overall Mediterranean diet have been reported to have higher intake of vitamin D, suggesting that other components may contribute to total vitamin D intake [45,46,47]. However, low consumption of dairy products and eggs (typical of the Mediterranean diet) does not allow reaching the suggested dietary intake of vitamin D [48]. Regardless of the high availability of sunshine, low serum 25(OH)D levels have been reported in adults in southern European countries, confirming the results obtained in the present study [49].

In the present study, there was a significant difference in vitamin E intake between men and women (the latter had lower intake), despite only a minority of both (about 10%) meeting the criteria for adequate consumption. At the European level, selenium deficiency was found in less than 10% of adults in northern Europe (Finland and the Netherlands), while in Italy and other northern European countries it was above 30% [40]. Vitamin E intake varied greatly in Europe, ranging from 6.5 mg/ day and 7.2 mg/day in women and men, respectively, living in Denmark to 11.9 mg/ day and 13.7 mg/ day in women and men, respectively, living in the Netherlands; the contribution from dietary supplements among adults, particularly women, was substantial in the aforementioned countries [50]. Recent mechanistic studies indicate that vitamin E has unique antioxidant and anti-inflammatory properties that may play a role in preventing metabolic disorders and chronic diseases [51]. Consistently with mechanistic findings, animal and human studies show that vitamin E may be helpful in preventing inflammation-associated diseases [52]. From a clinical point of view, vitamin E may decrease the blood levels of C-reactive protein (CRP), a marker of chronic inflammation that has a major role in the etiology of chronic disease [53]. Major food contributors of vitamin E are fruits and vegetables, which have been reported to be highly consumed in this sample. However, certain sources of vitamin E (such as avocado, papaya, etc.) are not commonly consumed in the Mediterranean area. In contrast, almonds are the most typical nut consumed in Sicily that is high in vitamin E; moderate consumption of nuts may play a role in the benefits associated with the Mediterranean diet, as it has been suggested to be a healthy food for a successful aging [54,55].

Among the vitamin B group, it is noteworthy to underline that less than half of the sample met the recommendations for vitamin B9 (folic acid). These estimates are in line with most European countries, where vitamin B9 intake ranged from 376 mg/day in the UK to 212 mg/g in the Dutch adult male population; the distribution of average consumption among women was slightly different, as women in the UK consumed relatively low amounts of vitamin B9 compared to other countries, while the highest intake was reported for the Portuguese and Italian adult female population [40]. Vitamins involved in one-carbon metabolism (including B9 and B12) have a major role during pregnancy for the developing fetus, preventing major congenital malformations such as neural tube defects [56,57]. Vitamin B9 has been studied for cancer prevention, as gene-nutrient interaction between the genes in the folate metabolic pathway and dietary folate availability have shown that mutations in genes of folate metabolism alter individual susceptibility to certain childhood cancers [58]. Moreover, in vitro and in vivo studies have shown that vitamin B9 may improve vascular endothelial function, thereby preventing the progression of CVD in individuals with overt disease or elevated CVD risk [59]. Particularly for women of childbearing age, deficient intake can have serious consequences for their offspring. Although it was not significant, folate intake was higher in this group compared to the total group of women; however, women had lower intake than men in all age groups. There is evidence that vitamin B9 intake is generally low in adult populations and that food fortification and supplement intake are needed to reach the suggested amount [60]. As previously reported, the sample had a relatively high intake of vegetables and legumes, which may provide adequate amounts of several phytochemicals and antioxidant compounds [61,62]. However, concerning vitamin B9, common dietary intake seems to be insufficient to reach the amount suggested, and supplementation with synthetic products may not be equivalent to consuming natural sources of vitamins (including vitamin B9) [63]. Nevertheless, vitamin B9 supplementation has been demonstrated to protect against neural tube defects and CVD [64,65,66]. Thus, information campaigns on folic acid availability and preferences would be of benefit for the general population and have large-scale public health implications in preventing neurological conditions in newborns [67]. 

Regarding other micronutrients and minerals, in this study we found that the nutritional recommendations were adequate overall. However, less than half of the sample met the recommendations for sodium, potassium, and calcium intake. High consumption of sodium and low consumption of potassium is a widespread issue all around the world: the mean global sodium intake has been estimated to be 3.95 g/day, with the highest in Eastern (mean >4.2 g/day) and Central (3.9–4.2 g/day) Europe; among European countries, Italy scored among the highest [68]. Regarding potassium intake, lower consumption has been reported in Germany, Sweden, and southern Italy [69]. Similarly, Italy is among the countries with the lowest consumption of calcium, while Northern European countries have the highest [70]. The potential effects of calcium on human health are matched with the role of vitamin D and have been discussed previously. The relationship between sodium and potassium intake and blood pressure levels is relatively well understood, while further consideration should be given to their potential role in endothelial cell dysfunction, progression of albuminuria and kidney disease, and cardiovascular disease–related morbidity and mortality [71]. It has been demonstrated that globally, 1.65 million annual deaths related to CVD were attributed to inadequate sodium intake [72]. In contrast, potassium per se has been not univocally reported as beneficial for health [73], while sodium-to-potassium ratio has been suggested as a potential marker for metabolic risk disorders [74,75,76]. A major dietary source of sodium has been commonly reported to be “added salt” and processed meat [77], but in this sample we found that the main contributor of sodium intake was grains. This is not surprising, considering the cultural heritage of the southern Mediterranean population, with production of pasta and bread; the latter has to be considered responsible for the aforementioned sodium intake. However, a slow abandonment of traditional foods for a “Westernized” diet has also been noted in this area [78], and the introduction of salted snacks (such as baked goods and crackers) in the diet may significantly contribute to total sodium intake, especially among the younger population.

The results presented in this study should be considered in light of some limitations. Comparing between nutritional surveys is a great challenge due to different methodologies used in various studies. Specifically, studies using 24 h recalls usually reported lower micronutrient intake than studies using food diaries and FFQs. Moreover, the number of items included in the FFQs may also affect the reporting of micronutrient intake. It is clear that an ideal method does not exist and both methodologies are widely used in nutritional epidemiology. However, a certain degree of under- and overestimation for dietary recalls and FFQs, respectively, should be taken into account.

## 5. Conclusions

In conclusion, despite meeting the national and European dietary recommendations for most micronutrients and minerals, there is evidence that the southern Italian population has low intake of vitamin D and calcium, vitamin E, and potassium and high intake of sodium. Considering the body of evidence on the beneficial and detrimental effects of the aforementioned dietary components, population-wide campaigns are needed in order to raise awareness of such dietary issues as an essential public health effort to prevent chronic noncommunicable diseases.

## Figures and Tables

**Table 1:** Total, sex-, and age-specific consumption of dietary vitamins for the participants of the Mediterranean Healthy Eating, Ageing, and Lifestyle (MEAL) study (*n* = 1838). * Denotes significant difference between sex (*p* < 0.05); † denotes significant difference between age groups. SE, standard error.

	Total			<20 years			20–50			50–70			>70 years		
	*n*	Mean (SE)	Median (Range)	*n*	Mean (SE)	Median (Range)	*n*	Mean (SE)	Median (Range)	*n*	Mean (SE)	Median (Range)	*n*	Mean (SE)	Median (Range)
Vitamin A															
Total	1838	868.72 (10.00)	776.04 (152.21, 4949.53)	53	930.31 (65.40)	842.47 (256.18, 2743.98)	963	873.19 (14.84)	770.37 (152.21, 4949.53)	597	873.40 (16.15)	785.75 (192.61, 2832.82)	225	822.69 (23.56)	759.64 (194.54, 2886.16)
Men	772	887.26 (16.22)	791.16 (172.82, 4949.53)	30	959.30 (61.61)	873.31 (417.78, 1772.20)	384	885.03 (25.90)	757.77 (172.82, 4949.53)	265	879.78 (24.49)	796.64 (231.20, 2331.72)	93	894.57 (38.26)	859.58 (353.52, 2263.34)
Women	1066	855.30 (12.60)	768.46 (152.21, 3211.70)	23	892.51 (129.05)	714.87 (256.18, 2743.98)	579	865.34 (17.74)	778.92 (152.21, 3211.70)	332	868.31 (21.50)	780.27 (192.61, 2832.82)	132	772.05 (29.09) *	722.17 (194.54, 2886.16)
Vitamin B1															
Total	1838	1.77 (0.02)	1.61 (0.41, 8.79)	53	1.91 (0.10)	1.81 (0.93, 4.05)	963	1.80 (0.03)	1.63 (0.41, 8.79)	597	1.75 (0.03)	1.59 (0.42, 6.11)	225	1.63 (0.04) †	1.52 (0.41, 4.50)
Men	772	1.82 (0.03)	1.63 (0.53, 8.79)	30	2.09 (0.12)	2.12 (1.03, 3.70)	384	1.87 (0.05)	1.65 (0.64, 8.79)	265	1.76 (0.05)	1.58 (0.53, 5.75)	93	1.69 (0.07)	1.54 (0.62, 4.50)
Women	1066	1.73 (0.02) *	1.59 (0.41, 6.11)	23	1.67 (0.15) *	1.48 (0.93, 4.05)	579	1.75 (0.03) *	1.61 (0.41, 5.74)	332	1.75 (0.04)	1.60 (0.42, 6.11)	132	1.59 (0.05)	1.49 (0.41, 4.42)
Vitamin B2															
Total	1838	2.24 (0.02)	2.06 (0.48, 10.44)	53	2.36 (0.12)	2.16 (0.97, 5.00)	963	2.27 (0.03)	2.08 (0.48, 10.44)	597	2.23 (0.04)	2.03 (0.48, 7.28)	225	2.14 (0.05)	2.00 (0.48, 5.12)
Men	772	2.29 (0.04)	2.05 (0.87, 10.44)	30	2.55 (0.14)	2.39 (1.29, 4.73)	384	2.33 (0.06)	2.08 (0.96, 10.44)	265	2.22 (0.06)	1.99 (0.87, 7.05)	93	2.19 (0.08)	2.10 (0.94, 5.12)
Women	1066	2.21 (0.03)	2.06 (0.48, 7.28)	23	2.11 (0.19)	1.99 (0.97, 5.00)	579	2.22 (0.04)	2.08 (0.48, 6.57)	332	2.24 (0.05)	2.06 (0.48, 7.28)	132	2.10 (0.06)	1.99 (0.48, 4.98)
Vitamin B3															
Total	1838	21.96 (0.19)	20.78 (4.13, 78.42)	53	20.90 (0.80)	19.94 (4.13, 38.57)	963	22.00 (0.27)	20.68 (7.44, 71.83)	597	22.32 (0.34)	21.22 (6.99, 78.42)	225	21.11 (0.40)	20.40 (8.18, 45.74)
Men	772	22.28 (0.31)	20.81 (4.13, 78.42)	30	21.21 (1.05)	21.16 (4.13, 32.47)	384	22.32 (0.46)	20.61 (8.23, 71.83)	265	22.67 (0.58)	21.07 (6.99, 78.42)	93	21.38 (0.62)	20.84 (9.81, 45.74)
Women	1066	21.73 (0.23)	20.77 (7.20, 65.67)	23	20.49 (1.24)	19.09 (14.28, 38.57)	579	21.79 (0.33)	20.68 (7.44, 65.67)	332	22.04 (0.39)	21.42 (7.20, 50.48)	132	20.93 (0.53)	20.16 (8.18, 39.88)
Vitamin B6															
Total	1838	2.55 (0.02)	2.43 (0.43, 8.46)	53	2.63 (0.12)	2.52 (0.43, 5.36)	963	2.55 (0.03)	2.39 (1.04, 8.46)	597	2.57 (0.04)	2.46 (0.84, 7.19)	225	2.49 (0.05)	2.40 (1.07, 4.76)
Men	772	2.60 (0.03)	2.46 (0.43, 8.46)	30	2.75 (0.16)	2.57 (0.43, 4.95)	384	2.59 (0.05)	2.42 (1.08, 8.46)	265	2.61 (0.06)	2.48 (1.03, 7.19)	93	2.61 (0.08)	2.52 (1.29, 4.76)
Women	1066	2.51 (0.03) *	2.40 (0.84, 6.52)	23	2.48 (0.19)	2.29 (1.35, 5.36)	579	2.52 (0.04)	2.37 (1.04, 6.52)	332	2.54 (0.04)	2.46 (0.84, 5.10)	132	2.41 (0.06) *	2.36 (1.07, 4.41)
Vitamin B9															
Total	1838	391.50 (3.86)	366.83 (61.31, 2535.14)	53	390.56 (20.41)	364.10 (61.31, 808.99)	963	395.44 (5.88)	363.44 (83.83, 2535,14)	597	388.86 (6.13)	374.15 (88.26, 1471.10)	225	381.84 (8.59)	369.55 (100.34, 829.71)
Men	772	401.17 (6.48)	370.70 (61.31, 2535.14)	30	409.11 (25.12)	406.79 (61.31, 745.20, 366.25)	384	402.70 (10.23)	370.06 (142.01, 2535.14)	265	398.02 (10.33)	363.36 (111.22, 1471.10)	93	401.23 (13.68)	378.32 (202.42, 810.86)
Women	1066	384.49 (4.71) *	364.69 (83.83, 1378.24)	23	366.35 (33.72)	319.99 (137.33, 808.99)	579	390.62 (7.05)	356.29 (83.83, 1378.24)	332	381.54 (7.31)	376.80 (88.26, 925.62)	132	368.17 (10.91)	357.82 (100.34, 829.71)
Vitamin B12															
Total	1838	6.18 (0.10)	5.50 (0.57, 120.52)	53	6.40 (0.37)	6.15 (2.33, 14.92)	963	6.23 (0.17)	5.36 (0.57, 120.52)	597	6.17 (0.14)	5.58 (1.59, 36.02)	225	5.92 (0.18)	5.53 (1.24, 19.68)
Men	772	6.45 (0.21)	5.56 (1.24, 120.52)	30	6.48 (0.53)	5.88 (2.33, 14.92)	384	6.47 (0.37)	5.29 (2.00, 120.52)	265	6.54 (0.26)	5.80 (1.62, 36.02)	93	6.15 (0.29)	5.69 (1.24, 19.68)
Women	1066	5.98 (0.10) *	5.40 (0.57, 31.75)	23	6.29 (0.53)	6.33 (2.92, 12.07)	579	6.08 (0.14)	5.47 (0.57, 31.75)	332	5.87 (0.15) *	5.30 (1.59, 16.52)	132	5.76 (0.22)	5.41 (1.50, 14.60)
Vitamin C															
Total	1838	158.09 (2.29)	132.65 (5.29, 1097.80)	53	151.40 (9.94)	139.84 (20.96, 308.49)	963	162.11 (3.53)	132.63 (5.29, 1097.80)	597	158.22 (3.62)	134.94 (6.09, 569.98)	225	142.15 (4.67) †	125.81 (17.67, 378.71)
Men	772	164.10 (3.78)	135.84 (12.73, 1097.80)	30	167.32 (13.70)	166.42 (20.96, 308.49)	384	165.47 (6.12)	131.28 (24.88, 1097.80)	265	163.82 (5.76)	136.48 (12.73, 569.98)	93	158.16 (7.62)	141.57 (47.61, 366.63)
Women	1066	153.74 (2.83) *	129.83 (5.29, 700.31)	23	130.62 (13.46)	107.79 (44.12, 297.64)	579	159.88 (4.25)	134.02 (5.29, 700.31)	332	153.74 (4.59)	132.82 (6.09, 500.62)	132	130.87 (5.70) *	120.41 (17.67,378.71)
Vitamin D															
Total	1838	5.16 (0.10)	3.93 (0.20, 56.42)	53	5.47 (0.43)	4.84 (0.60, 13.64)	963	5.02 (0.15)	3.78 (0.20, 56.42)	597	5.37 (0.19)	4.01 (0.56, 45.67)	225	5.09 (0.27)	4.19 (0.58, 30.24)
Men	772	5.19 (0.18)	3.93 (0.56, 56.42)	30	5.11 (0.60)	4.28 (0.60, 13.64)	384	4.89 (0.25)	3.72 (0.56, 56.42)	265	5.61 (0.32)	4.28 (0.56, 45.67)	93	5.30 (0.48)	4.40 (0.58, 30.24)
Women	1066	5.13 (0.13)	3.91 (0.20, 34.12)	23	5.94 (0.62)	5.62 (2.14, 12.96)	579	5.11 (0.18)	3.83 (0.20, 34.12)	332	5.19 (0.22)	3.79 (0.57, 28.71)	132	4.94 (0.32)	4.15 (0.63, 24.90)
Vitamin E															
Total	1838	8.66 (0.08)	8.04 (1.47, 31.93)	53	8.86 (0.42)	8.38 (2.68, 18.44)	963	8.75 (0.11)	8.02 (1.66, 25.56)	597	8.70 (0.14)	8.10 (1.47, 31.93)	225	8.07 (0.17)	7.85 (3.16, 18.36)
Men	772	8.85 (0.13)	8.12 (2.68, 31.93)	30	9.38 (0.63)	8.62 (2.68, 18.44)	384	8.79 (0.19)	7.77 (3.28, 25.56)	265	9.05 (0.24)	8.44 (3.17, 31.93)	93	8.35 (0.27)	8.11 (4.43, 18.36)
Women	1066	8.51 (0.09) *	8.03 (1.47, 22.08)	23	8.18 (0.50)	8.29 (3.72, 15.77)	579	8.72 (0.14)	8.14 (1.66, 22.08)	332	8.43 (0.16) *	7.94 (1.47, 18.42)	132	7.87 (0.21)	7.77 (3.16, 15.32)

**Table 2:** Total, sex-, and age–specific consumption of dietary minerals for the participants of the MEAL study (*n* = 1838). * Denotes significant difference between sex (*p* <0.05).

	Total			<20 years			20–50			50–70			>70 years		
	*n*	Mean (SE)	Median (Range)	*n*	Mean (SE)	Median (Range)	*n*	Mean (SE)	Median (Range)	*n*	Mean (SE)	Median (Range)	*n*	Mean (SE)	Median (Range)
Calcium															
Total	1838	800.35 (7.76)	744.24 (169.71, 3109.93)	53	842.68 (44.12)	784.64 (304.55, 1662.32)	963	801.60 (11.36)	734.79 (171.97, 3109.93)	597	794.99 (12.61)	747.21 (169.71, 2294.96)	225	799.28 (20.69)	773.11 (172.00, 1839.22)
Men	772	803.27 (12.32)	736.34 (224.53, 3109.93)	30	900.43 (53.54)	861.76 (507.68, 1662.32)	384	801.99 (18.92)	705.14 (245.67, 3109.93)	265	785.15 (19.29)	733.38 (224.53, 2294.96)	93	828.90 (31.92)	813.83 (350.52, 1839.22)
Women	1066	798.23 (9.97)	753.51 (169.71, 2186.26)	23	767.36 (72.23)	653.88 (304.55, 1554.25)	579	801.34 (14.14)	749.32 (171.97, 2186.26)	332	802.84 (16.65)	767.44 (164.71, 2015.75)	132	778.40 (27.12)	733.15 (172.00, 1817.71)
Sodium															
Total	1838	2847.07 (25.59)	2689.04 (737.51, 9469.51)	53	2823.07 (139.92)	2940.59 (796.60, 5317.25)	963	2910.60 (36.70)	2774.86 (737.51, 9391.07)	597	2792.70 (45.29)	2625.90 (834.83, 9469.51)	225	2725.10 (58.25)	2613.69 (829.26, 5251.12)
Men	772	2924.44 (41.41)	2727.70 (796.60, 9469.51)	30	3027.64 (190.91)	3185.84 (796.60, 5317.25)	384	2970.18 (61.42)	2779.33 (870.96, 9273.63)	265	2906.64 (71.29)	2692.50 (903.42, 9469.51)	93	2753.03 (93.36)	2655.48 (1167.12, 5199.53)
Women	1066	2791.04 (32.28)	2661.75 (737.51, 9391.07)	23	2556.23 (195.57)	2453.54 (1097.52, 4746.73	579	2871.08 (45.44)	2766.61 (737.51, 9391.07)	332	2701.76 (57.88)	2567.36 (834.83, 7747.75)	132	2705.43 (74.62)	2597.31 (829.26, 5251.12)
Iron															
Total	1838	15.27 (0.14)	14.40 (4.13, 84.06)	53	15.13 (0.66)	14.63 (4.13, 27.90)	963	15.41 (0.21)	14.33 (4.83, 84.06)	597	15.24 (0.22)	14.60 (4.42, 50.81)	225	14.76 (0.30)	13.96 (6.75, 31.34)
Men	772	15.59 (0.23)	14.53 (4.13, 84.06)	30	15.64 (0.86)	15.30 (4.13, 25.85)	384	15.68 (0.36)	14.45 (5.54, 84.06)	265	15.55 (0.37)	14.59 (4.42, 50.81)	93	15.34 (0.49)	14.47 (6.75, 31.34)
Women	1066	15.03 (0.17) *	14.26 (4.83, 41.09)	23	14.47 (1.02)	12.58 (7.72, 27.90)	579	15.23 (0.25)	14.26 (4.83, 41.09)	332	14.99 (0.27)	14.67 (5.25, 35.40)	132	14.35 (0.39)	13.50 (7.07, 29.99)
Magnesium															
Total	1838	392.99 (3.24)	372.88 (109.85, 1665.14)	53	382.56 (16.08)	364.41 (109.85, 699.45)	963	395.21 (4.90)	369.92 (147.60, 1665.14)	597	394.57 (5.29)	385.25 (145.91, 1089.24)	225	381.76 (7.00)	366.82 (152.02, 709.36)
Men	772	396.92 (5.37)	374.76 (109.85, 1665.14)	30	392.08 (22.05)	379.14 (109.85, 699.45)	384	399.62 (8.48)	372.31 (158.30, 1665.14)	265	395.51 (8.63)	385.01 (149.58, 1089.24)	93	391.34 (10.68)	375.45 (162.04, 654.69)
Women	1066	390.15 (4.00)	372.08 (145.91, 954.25)	23	370.15 (23.67)	348.24 (246.17, 687.34)	579	392.29 (5.90)	369.29 (147.60, 954.25)	332	393.83 (6.57)	386.78 (145.91, 863.13)	132	375.01 (9.24)	363.79 (152.02, 709.36)
Potassium															
Total	1838	3653.59 (32.01)	3446.04 (812.57, 15693.66)	53	3731.19 (176.32)	3690.30 (812.57, 7852.01)	963	3674.80 (47.74)	3415.25 (872.14, 15693.66)	597	3669.38 (52.85)	3531.47 (1050.23, 11778.63)	225	3502.63 (72.19)	3375.85 (1206.18, 8039.89)
Men	772	3732.00 (53.25)	3509.80 (812.57, 15693.66)	30	3902.19 (228.04)	3795.98 (812.57, 7156.95)	384	3712.12 (82.16)	3455.26 (1574.87, 15693.66)	265	3757.96 (88.36)	3553.17 (1642.82, 11778.63)	93	3685.19 (109.37)	3626.06 (1775.75, 6615.19)
Women	1066	3596.81 (39.41) *	3397.32 (872.14, 9165.64)	23	3508.15 (275.53)	2998.47 (1799.16, 7852.01)	579	3650.05 (57.78)	3395.22 (872.14, 9165.64)	332	3598.68 (63.55)	3507.39 (1050.23, 8028.33)	132	3374.00 (94.69) *	3213.99 (1206.18, 8039.89)
Selenium															
Total	1838	103.07 (1.06)	93.91 (21.56, 431.10)	53	100.74 (5.07)	95.43 (21.56, 212.36)	963	102.42 (1.53)	91.61 (23.14, 431.10)	597	104.97 (1.82)	96.23 (24.69, 289.43)	225	101.36 (2.58)	94.02 (32.53, 239.21)
Men	772	103.96 (1.69)	91.84 (21.56, 431.10)	30	99.37 (5.90)	95.65 (21.56, 171.70)	384	104.57 (2.59)	90.70 (25.48, 431.10)	265	105.10 (2.78)	93.80 (24.69, 276.54)	93	99.69 (3.95)	90.64 (33.27, 239.21)
Women	1066	102.43 (1.35)	95.14 (23.14, 289.43)	23	102.52 (8.93)	95.43 (49.01, 212.36)	579	101.00 (1.88)	92.31 (23.14, 289.09)	332	104.86 (2.42)	97.79 (40.31, 289.43)	132	102.53 (3.42)	97.08 (32.53, 228.10)
Zinc															
Total	1838	12.19 (0.11)	11.48 (3.66, 54.05)	53	12.41 (0.51)	11.89 (5.15, 22.17)	963	12.27 (0.16)	11.37 (3.67, 54.05)	597	12.19 (0.18)	11.69 (3.67, 39.48)	225	11.76 (0.24)	11.30 (3.66, 22.69)
Men	772	12.40 (0.18)	11.51 (4.54, 54.05)	30	12.73 (0.68)	12.24 (5.15, 22.17)	384	12.39 (0.28)	11.28 (5.04, 54.05)	265	12.41 (0.28)	11.73 (4.54, 39.48)	93	12.26 (0.38)	11.62 (5.52, 22.69)
Women	1066	12.04 (0.13)	11.47 (3.66, 31.84)	23	12.00 (0.77)	11.47 (6.41, 20.56)	579	12.19 (0.18)	11.46 (3.67, 31.84)	332	12.02 (0.22)	11.61 (3.67, 26.81)	132	11.40 (0.30)	11.01 (3.66, 20.56)

**Table 3:** Number and percentage of individuals meeting the European recommendations for mineral and vitamin intake in the MEAL study (*n* = 1838).

	Total (*n* = 1851)		Men (*n* = 772)		Women (*n* = 1066)	
	Yes, % (*n*)	No, % (*n*)	Yes, % (*n*)	No, % (*n*)	Yes, % (*n*)	No, % (*n*)
EFSA						
Calcium	24.0 (442)	76.0 (1397)	24.6 (190)	75.4 (582)	23.6 (252)	76.4 (814)
Iron	64.4 (1185)	35.6 (654)	79.1 (611)	20.9 (161)	53.8 (574)	46.2 (492)
Magnesium	66.9 (1230)	33.1 (609)	57.6 (445)	42.4 (327)	73.6 (785)	26.4 (281)
Potassium	48.1 (884)	51.9 (955)	50.5 (390)	49.5 (382)	46.3 (494)	53.7 (572)
Selenium	75.8 (1394)	24.2 (445)	76.3 (589)	23.7 (183)	75.4 (804)	24.6 (262)
Vitamin A	60.1 (1106)	39.9 (733)	53.6 (414)	46.4 (358)	64.9 (692)	35.1 (374)
Vitamin B1	100 (1839)	–	100 (772)	–	100 (1066)	–
Vitamin B2	77.0 (1416)	23.0 (423)	78.5 (606)	21.5 (166)	75.9 (809)	24.1 (257)
Vitamin B3	100 (1839)	–	100 (772)	–	100 (1066)	–
Vitamin B6	87.1 (1601)	12.9 (238)	86.8 (670)	13.2 (102)	87.3 (931)	12.7 (135)
Vitamin B9	60.3 (1108)	39.7 (731)	62.3 (481)	37.7 (291)	58.8 (627)	41.2 (439)
Vitamin B12	75.4 (1387)	24.6 (452)	77.2 (596)	22.8 (176)	74.1 (790)	25.9 (276)
Vitamin C	70.5 (1296)	29.5 (543)	65.5 (506)	34.5 (266)	74.1 (790)	25.9 (276)
Vitamin D	3.2 (58)	96.8 (1781)	3.2 (25)	96.8 (747)	3.1 (33)	96.9 (1033)
Vitamin E	14.8 (273)	85.2 (1566)	10.5 (81)	89.5 (691)	18.0 (192)	82.0 (874)
Zinc	62.4 (1148)	37.6 (691)	47.7 (368)	52.3 (404)	73.2 (780)	26.8 (286)

**Table 4:** Number and percentage of individuals meeting the Italian recommendations for mineral and vitamin intake in the MEAL study (*n* = 1838).

	Total (*n* = 1851)		Men (*n* = 772)		Women (*n* = 1066)	
	Yes, % (*n*)	No, % (*n*)	Yes, % (*n*)	No, % (*n*)	Yes, % (*n*)	No, % (*n*)
LARN						
Calcium	20.1 (370)	79.9 (1469)	20.5 (158)	79.5 (614)	19.9 (212)	80.1 (854)
Sodium	91.6 (1695)	7.8 (144)	93.3 (720)	6.7 (102)	91.4 (974)	8.6 (92)
Iron	51.2 (942)	48.8 (897)	86.8 (670)	13.2 (102)	25.5 (272)	74.5 (794)
Magnesium	90.4 (1663)	9.6 (176)	90.4 (698)	9.6 (74)	90.5 (965)	9.5 (101)
Potassium	35.8 (658)	64.2 (1181)	37.6 (290)	62.4 (484)	34.5 (368)	65.5 (698)
Selenium	90.5 (1665)	9.5 (174)	92.1 (711)	7.9 (61)	89.4 (953)	10.6 (113)
Vitamin A	66.7 (1226)	33.3 (613)	61.4 (474)	38.6 (298)	70.5 (752)	29.5 (314)
Vitamin B1	80.2 (1475)	19.8 (364)	78.1 (603)	21.9 (169)	81.8 (872)	18.2 (194)
Vitamin B2	85.0 (1564)	15.0 (275)	78.5 (606)	21.5 (166)	89.9 (958)	10.1 (108)
Vitamin B3	68.7 (1264)	31.3 (575)	69.4 (536)	30.6 (236)	68.3 (728)	31.7 (338)
Vitamin B6	97.5 (1793)	2.5 (46)	97.9 (756)	2.1 (16)	97.2 (1036)	2.8 (30)
Vitamin B9	41.0 (754)	59.0 (1085)	43.0 (332)	57.0 (440)	39.6 (422)	60.4 (644)
Vitamin B12	96.2 (1770)	3.8 (69)	98.3 (759)	17 (13)	94.7 (1010)	5.3 (56)
Vitamin C	75.1 (1382)	24.9 (457)	68.8 (531)	31.2 (241)	79.8 (851)	20.2 (215)
Vitamin D	3.1 (57)	96.9 (1782)	3.2 (25)	96.8 (747)	3.0 (32)	97.0 (1034)
Vitamin E	11.5 (211)	88.5 (1628)	10.5 (81)	89.5 (691)	12.2 (130)	87.8 (936)
Zinc	62.9 (1157)	37.1 (682)	45.1 (348)	54.9 (424)	75.9 (809)	24.1 (257)
